# Periostin Plasma Levels and Changes on Physical and Cognitive Capacities in Community-Dwelling Older Adults

**DOI:** 10.1093/gerona/glac226

**Published:** 2022-11-14

**Authors:** Juan Luis Sánchez-Sánchez, Isabelle Ader, Yannick Jeanson, Valérie Planat-Benard, Bruno Vellas, Louis Casteilla, Philipe de Souto-Barreto

**Affiliations:** Gérontopôle de Toulouse, Institut du Vieillissement, Centre Hospitalier Universitaire de Toulouse, Toulouse, France; Department of Health Sciences, Universidad Pública de Navarra, Pamplona, Spain; Institut RESTORE, Université de Toulouse, CNRS U-5070, EFS, ENVT, INSERM U1301, France; Institut RESTORE, Université de Toulouse, CNRS U-5070, EFS, ENVT, INSERM U1301, France; Institut RESTORE, Université de Toulouse, CNRS U-5070, EFS, ENVT, INSERM U1301, France; Gérontopôle de Toulouse, Institut du Vieillissement, Centre Hospitalier Universitaire de Toulouse, Toulouse, France; CERPOP, INSERM 1295, Université de Toulouse, UPS, Toulouse, France; Institut RESTORE, Université de Toulouse, CNRS U-5070, EFS, ENVT, INSERM U1301, France; Gérontopôle de Toulouse, Institut du Vieillissement, Centre Hospitalier Universitaire de Toulouse, Toulouse, France; CERPOP, INSERM 1295, Université de Toulouse, UPS, Toulouse, France

**Keywords:** Aging biomarker, Extracellular matrix, Healthy aging

## Abstract

Periostin, involved in extracellular matrix development and support, has been shown to be elevated in senescent tissues and fibrotic states, transversal signatures of aging. We aimed to explore associations between plasma periostin and physical and cognitive capacity evolution among older adults. Our hypothesis was that higher levels of plasma periostin will be associated with worse physical and mental capacities along time. Analyses included 1 096 participants (mean age = 75.3 years ± 4.4; 63.9% women) from the Multidomain Alzheimer Preventive Trial. Periostin levels (pg/mL) were measured in plasma collected at year 1. Periostin was used in continuous variable, and as a dichotomous variable highest quartile (POSTN+) versus lowest 3 quartiles (POSTN−) were used. Outcomes were measured annually over 4 years and included: gait speed (GS), short physical performance battery (SPPB) score, 5-times sit-to-stand test (5-STS), and handgrip strength (HS) as physical and cognitive composite *z*-score (CCS) and the Mini-Mental State Examination (MMSE) as cognitive endpoints. Plasma periostin as a continuous variable was associated with the worsening of physical and cognitive capacities over 4 years of follow-up, specifically the SPPB score, the 5-STS, and CCS in full-adjusted models. POSTN+ was associated with worse evolution in the physical (GS: [β = −0.057, 95% confidence interval (CI) = −0.101, −0.013], SPPB score [β = −0.736, 95% CI = −1.091, −0.381], 5-STS [β = 1.681, 95% CI = 0.801, 2.561]) as well as cognitive (CCS [β = −0.215, 95% CI = −0.335, −0.094]) domains compared to POSTN− group. No association was found with HS or the MMSE score. Our study showed for the first time that increased plasma periostin levels were associated with declines in both physical and cognitive capacities in older adults over a 4-year follow-up. Further research is needed to evaluate whether periostin might be used as a predictive biomarker of functional decline at an older age.

## Introduction

Population aging is a worldwide phenomenon that constitutes a societal challenge due to the increased welfare and health care costs (1). In this sense, strategies oriented at the expansion of health span are needed to assist in reducing this burden (2). Recently, World Health Organization has proposed a function-centered approach of older adults’ health (3). Under this framework, the identification of biomarkers that can predict the progression in physical and cognitive capacities (key components of functional ability) is critical (a) to predict adverse outcomes in late life, (b) to implement interventions aiming at promoting healthy aging, and (c) to monitor the response to these interventions (4).

Physical and cognitive capacities are the reflection of the integrative activity of different physiological systems, resulting from the function and architecture of integrated organs (5). Their impairments are secondary to the parallel dysregulation of multiple physiological systems (6). Therefore, measurable markers of physiological aging should be transverse, reflecting the integrative function of multiple systems over single-system biomarkers (7).

In the search of common elements to the whole organism, the supportive stromal cell compartment, especially mesenchymal stem cells (MSC) and their derivates, appear as basic transverse elements, given that they are the source and driver for every tissue architecture and repair mechanisms (8). As MSC derivates, fibroblasts and myofibroblasts produce and remodel the extracellular matrix (ECM). They are responsible for tissue structure and cell communication (9). In addition, they produce matricellular proteins (MPs), nonstructural components of the ECM that modify cell behavior through cell surface receptors such as integrins and exhibit diverse functions that exert control over development, pathology, and tissue healing processes (9–13). At the tissular level, aging is characterized by hardening, overgrowth, and scarring of various tissues by the accumulation of ECM components (14–17), leading to fibrosis, stiffness, dysfunctional remodeling, and altered ECM-mediated cellular cross-talk (18,19).

Among MPs, periostin (POSTN), also known as osteoblast-specific factor-2 (20), physiologically modulates ECM organization playing a unique role in the connection between the cells and the microenvironment that surrounds them (21) and translates signals between ECM and cell (22). Physiologically, it is involved in the normal development of teeth, bones, heart, and the kidney during embryonic development (20,23). However, it has also been shown to be elevated in profibrotic tissues (24,25), a signature of aging. In this environment, POSTN might contribute to tissues exhibiting harmful chronic inflammation and cellular senescence, resulting in loss of function, and eventually contributing to age-related diseases and subclinical changes associated with aging (26–30). Therefore, POSTN might be a candidate marker of whole-organism loss of physiological reserve and age-related organ functional impairment, which manifest themselves as reductions in physical and mental capacities, before overt clinical functional changes are detectable (31).

Altogether, this novel body of evidence supports the role of POSTN in tissue aging and inflammation, but to the best of our knowledge, no study has explored the potential association between POSTN and functional outcomes in aging humans so far.

Therefore, our study aims to explore whether plasma POSTN levels are longitudinally associated with physical and mental functions in community-dwelling older adults. We hypothesize that higher levels of plasma POSTN will be associated with worse physical and mental capacities along time.

## Method

This observational prospective analysis uses data from the Multidomain Alzheimer Preventive Trial (MAPT, ClinicalTrials.gov [NCT00672685]), a randomized, multicenter, placebo-controlled trial conducted with community-dwelling older adults in France and Monaco. Participants were allocated into 4 groups, either receiving ω-3 polyunsaturated fatty acid (PUFA) supplementation, a multidomain intervention (based on cognitive training, nutritional counseling, and physical activity advice), both, or placebo. The intervention lasted for 3 years and was followed by an additional 2-year observational phase. Recruitment of participants started in May 2008 and ended in February 2011. Follow-up ended in April 2016.

A detailed description of the MAPT study can be found elsewhere (32,33). In summary, eligibility criteria comprised: age 70 years or older; not presenting major neurocognitive disorders, Mini-Mental State Examination (MMSE) score ≥24; presenting at least 1 of the following: spontaneous memory concern, inability to perform 1 instrumental activity of daily living, or slow usual-pace walking speed (<0.8 m/sec). Participants were not included if they declared the use of ω-3 PUFA supplements during the 6 months before inclusion.

The population of the present study was composed of 1 096 participants with data on plasma POSTN levels at MAPT 1-year follow-up visit, where blood-based biomarkers were first measured in the study. The present study followed the Strengthening the Reporting of Observational Studies in Epidemiology (STROBE) guideline (34).

### POSTN Measurements and Status

Nonfasting blood samples were collected at MAPT year 1 visit each visit and were stored at −80°C. Briefly, plasma POSTN was quantified by a single disposable microfluidic SimplePlex cartridge using the fully automated immunoassay platform, Ella (ProteinSimple/Bio-techne, San Jose, CA). The plasma samples were thawed on ice and diluted 1:10 in sample diluent (SD 13) and loaded into cartridges with relevant high and low control concentrates. Each protein channel contains 3 analyte-specific glass nanoreactors, which allows for each plasma sample to be run in triplicates for target protein samples. Cartridges include a built-in lot-specific standard curve for defined plasma protein. All steps in the procedure were run automatically by the instrument with no user activity. The obtained data were displayed as pg/mL and automatically calculated by the internal instrument software.

For analytical purposes, POSTN was used as a continuous variable but also as a binary variable. In the absence of cut-points to define plasma POSTN abnormal values, we used the quartiles in POSTN levels to define high values (POSTN+; Q4 >28 169 pg/mL and POSTN− [≤Q4] groups).

### Outcome Measures

Outcomes were assessed in the same visit in which blood periostin was measured (1-year follow-up visit in MAPT study, data collected before periostin measurement were not used); and annually for 4 years (24, 36, and 48-month follow-up visits in MAPT). Overall cognitive performance was assessed using: a composite cognitive score (CCS) (33) based on four tests (the 10 orientation items of the MMSE, the Digit Symbol Substitution Test [DSST], free and total recall of the Free and Cued Selective Reminding Test [FCSRT], and the Category Naming Test); and the MMSE score (35). CCS was computed as the mean *z*-score of the 4 domains, calculated using the baseline mean and SD values of the corresponding test.

The physical capacities were evaluated by the usual pace gait speed test (GS) (36), the Short Physical Performance Battery (SPPB) (37), the 5-repetition sit-to-stand test (5-STS) (38), and the maximal handgrip strength (HS) (39).

### Covariates

Covariates consisted of age, sex, body mass index (BMI; kg/m²), MAPT group allocation educational level, and inflammation (assessed by plasma levels of interleukin-6 in pg/mL [IL-6]). All confounders were measured at the 1-year MAPT visit.

### Statistical Analyses

Descriptive statistics (mean ± standard deviation [*SD*] or frequencies and percentages, as appropriate) were used for the characterization of the study population. Quantitative variables at baseline (the 1-year visit where plasma periostin levels were measured) were compared according to POSTN status by Student’s *t* tests, and categorical variables were compared using the χ ^2^ tests.

Linear mixed-effects (LME) regression analyses (with random intercept and random slope for each participant) were performed to determine associations between baseline plasma POSTN (either continuous or dichotomous) and the overtime evolution of the outcome measures. The LME models included the fixed effects of baseline plasma POSTN, time as categorical, their interaction, and potential confounders (model 1: unadjusted; model 2: model 1 + body mass index (BMI), MAPT randomization group and educational level; model 3: model 2 + IL-6). Statistical significance for all test was set at an alpha value of 0.05. All analyses were performed using Stata 14.0 software (Stata Corp LLC, College Station, TX).

## Results

### Characterization of the Sample

Table 1 shows the characteristics of the 1 096 participants included in the present analyses (65.33% of the MAPT whole sample). Differences at baseline (1-year visit, where POSTN was measured in blood) between people included in the present study and those not included are displayed in Supplementary Table 1. The mean POSTN was 24 235.16 ± 7 224.77 pg/mL (coefficient of variation: 1.429% ± 0.649%). Two hundred seventy-four participants were classified as POSTN+. These participants were significantly older and presented with a higher BMI, and lower GS, SPPB score, CCS, and MMSE scores. Median (IQR) follow-up was 3.4 ± 1.0 years (Table 1).

**Table 1.** Baseline Characteristics of the Sample

Characteristics	Whole Sample Total (*n* = 1096)	Low Periostin (≤28169 pg/mL) (*n* = 822)	High Periostin (>28169 pg/mL) (*n* = 274)**	T-Statistic
Women, no. (%)	700 (63.87%)	554 (67.40)	146 (53.28)***	
Age, year	75.31 (4.37)	74.96 (4.15)	76.35 (4.84)***	−4.5980
MAPT group allocation, no. (%)				
Omega 3 + MDI group	417 (24.84)	200 (24.33)	74 (27.01)	
Omega 3 group	422 (25.13)	1 888 (22.87)	78 (288.47)	
MDI group	420 (25.01)	215 (26.16)	62 (22.63)	
Control group	420 (25.01)	219 (26.64)	60 (21.90)	
Education, no. (%)				
No diploma	85 (5.17)	35 (4.34)	14 (5.15)	
Primary school certificate	286 (17.41)	130 (16.11)	48 (17.65)	
Secondary education	553 (33.66)	260 (32.22)	94 (34.56)	
High school diploma	242 (14.73)	124 (15.37)	44 (16.18)	
University level	477 (29.03)	258 (31.97)	72 (26.47)	
Body mass index^†^	26.22 (4.05)	26.38 (4.06)	25.73 (3.99)***	2.3286
Plasma interleukin-6 (pg/mL)	3.89 (12.20)	3.55 (6.01)	4.90 (22.05)	−1.5845
Gait speed (m/s)	1.09 (0.27)	1.10 (0.28)	1.06 (0.27)***	2.6754
SPPB score	10.68 (1.75)	10.82 (1.56)	10.24 (2.16)***	4.7239
SPPB-STS test (s)	11.51 (3.79)	11.46 (3.86)	11.67 (3.86)	−0.7645
Handgrip strength (kg)	26.73 (9.74)	26.56 (9.74)	27.24 (9.74)	−0.9616
CCS^‡^	0.03 (0.69)	0.09 (0.66)	−0.14 (0.74)***	4.6823
MMSE	28.07 (1.81)	28.14 (1.76)	27.85 (1.93)***	2.2855
FCSRT delayed recall	76.11 (10.53)	76.75 (10.09)	74.19 (11.58)***	3.4905

*Notes*: MDI = Multi Domain Intervention; MAPT = Multidomain Alzheimer Preventive Trial; m/s = meters per second; SPPB = short physical performance battery; STS = sit-to-stand test; kg = kilograms; CCS = composite cognitive score; MMSE = Mini-Mental State Examination; FCSRT = Free and Cued Selective Reminding test.

^†^Body mass index calculated as weight in kilograms divided by height in meters squared.

^‡^Based on the *z*-score of 4 cognitive tests (free and total recall of the Free and Cued Selective Reminding test, 10 MMSE orientation items, Digit Symbol Substitution Test, and Category Naming Test).

**High plasma periostin is defined as values in the fourth quartile.

***p < .001 based on Student’s *t* test or Pearson χ ^2^ test (between periostin groups).

### Associations Between Periostin and Capacities (Periostin as Continuous)

At the cross-sectional level, plasma POSTN showed negative significant associations with GS (β = −3.16e-06, 95% confidence interval [CI] = −5.43e-06, −8.77e-07) and the CCS (β = −1.05e-05, 95% CI = −1.62e-05, −4.81e-06) in the full-adjusted model (data not shown).

Longitudinally, higher POSTN levels were significantly associated with worse SPPB scores (β = −2.08e-05, 95% CI = −3.97e-05, −1.95e-06), a greater time to complete the 5-STS test (β = 8.44e-05, 95% CI = 3.48e-05, 1.34e-04; Table 2) and worse CCS score (β = −6.04e-06, 95% CI = −1.11e-05, −9.50e-07) in full-adjusted models (Table 3).

**Table 2.** Associations Between Periostin and Longitudinal Evolution in Physical Functions (Periostin as Continuous)

Parameters	Sample Size	Model 1				Model 2				Model 3			
		Time-point (year)	Coefficient	*p*	95% CI	Time-point (year)	Coefficient	*p*	95% CI	Time-point (year)	Coefficient	*p*	95% CI
Gait speed (m/s)	1 067	1	−6.46e-07	.531	−2.67e-06, 1.38e-06	1	−5.65e-07	.591	−2.63e-06, 1.50e-06	1	−5.60e-07	.595	−2.62e-06, 1.50e-06
		2	−4.24e-07	.693	−2.53e-06, 1.68e-06	2	−3.16e-07	.771	−2.44e-06, 1.81e-06	2	−3.05e-07	.778	−2.43e-06, 1.82e-06
		3	−5.29e-08	.965	−2.44e-06, 2.33e-06	3	7.02e-08	.954	−2.31e-06, 2.45e-06	3	8.22e-08	.946	−2.29e-06, 2.46e-06
		4	1.40e-07	.914	−2.39e-06, 2.67e-06	4	3.59e-07	.777	−2.13e-06, 2.85e-06	4	3.97e-07	.754	−2.09e-06, 2.88e-06
SPPB score	1 069	1	1.42e-05	.044	4.09e-07, 2.79e-05	1	1.47e-05	.039	7.07e-07, 2.87e-05	1	1.48e-05	.039	7.43e-07, 2.88e-05
		2	2.78e-06	.712	−1.2e-05, 1.75e-05	2	3.46e-06	.649	−1.14e-05, 1.83e-05	2	3.50e-06	.645	−1.14e-05, 1.84e-05
		3	−4.07e-06	.645	−2.14e-05, 1.32e-05	3	−3.61e-06	.682	−2.09e-05, 1.37e-05	3	−3.62e-06	.681	−2.09e-05, 1.36e-05
		4	−**2.19e-05**	**.025**	−**4.1e-05,** −**2.71e-06**	4	−**2.09e-06**	**.030**	−**3.98e-05, -2.03e-06**	4	−**2.08e-05**	**.031**	−**3.97e-05,** −**1.95e-06**
5-STS (s)	1 062	1	−9.91e-06	.589	−4.59 e-05, 2.6e-04	1	−1.11e-05	.550	−4.75e-05, 2.53e-05	1	−1.11e-05	.552	−4.75e-05, 2.54e-05
		2	2.22e-05	.254	−1.6e-05, 6.04e-05	2	2.08e-05	.291	−1.78e-05, 5.93e-05	2	2.08e-05	.290	−1.77e-05, 5.93e-05
		3	−7.10e-06	.755	−5.16e-05, 3.74e-05	3	−7.50e-06	.742	−5.22e-05, 3.72e-05	3	−7.91e-06	.729	−5.26e-05, 3.68e-05
		4	**8.76e-05**	**.001**	**3.8e-05, 1.37e-04**	4	**8.43e-05**	**.001**	**3.47e-05, 1.3e-04**	4	**8.44e-05**	**.001**	**3.48e-05, 1.34e-04**
Handgrip strength (kg)	1 090	1	−6.88e-06	.796	−5.89e-05, 4.52e-05	1	−6.33e-06	.814	−5.89e-05, 4.62e-05	1	−6.48e-06	.809	−5.88e-05, 4.61e-05
		2	1.52e-05	.577	−3.83e-05, 6.88e-05	2	1.52e-05	.579	−3.85e-05, 6.88e-05	2	1.49e-05	.583	−3.86e-05, 6.87e-05
		3	8.18e-07	.978	−5.79e-05, 5.95e-05	3	−1.35e-06	.963	−5.93e-05, 5.66e-05	3	−1.54e-06	.959	−5.95e-05, 5.64e-05
		4	1.89e-05	.551	−4.33e-05, 8.12e-05	4	1.48e-05	.631	−4.57e-05, 7.54e-05	4	1.48e-05	.632	−4.58e-05, 7.54e-05

*Notes*: Significant associations are in bold. CI = confidence interval; m/s = meters per second; SPPB = short physical performance battery; STS = sit-to-stand test; kg = kilograms.

**Table 3.** Associations Between Periostin and Longitudinal Evolution in Cognitive Capacities (Periostin as Continuous)

Parameters	Sample Size	Model 1				Model 2				Model 3			
		Time-point (year)	Coefficient	*p*	95% CI	Time-point (year)	Coefficient	*p*	95% CI	Time-point (year)	Coefficient	*p*	95% CI
CCS	1 067	1	−3.02e-06	.094	−6.56e-06, 5.12e-07	1	−3.07e-06	.090	−6.62e-06, 4.80e-07	1	−3.06e-06	.091	−6.62e-06, 4.90e-07
		2	−3.47e-06	.080	−7.36e-06, 4.20e-07	2	−3.40e-06	.087	−7.30e-06, 4.98e-07	2	−3.40e-06	.088	−7.30e-06, 5.08e-07
		3	−3.38e-06	.144	−7.90e-06, 1.15e-06	3	−3.22e-06	.164	−7.75e-06, 1.32e-06	3	−3.21e-06	.166	−7.75e-06, 1.33e-06
		4	**−6.14e-06**	**.018**	−**1.12e-05, -1.07e-06**	4	−**6.02e-06**	**.020**	−**1.11e-05,** −**9.40e-07**	4	−**6.04e-06**	**.020**	−**1.11e-05,** −**9.50e-07**
MMSE	1 069	1	1.15e-05	.130	−3.40e-06, 2.64e-05	1	0.000012	.119	−3.08e-06, 2.7e-05	1	1.2e-05	.119	−3.09e-06, 2.7e-05
		2	−2.33e-06	.768	−1.78e-05, 1.31e-05	2	−2.06e-06	.795	−1.76e-05, 1.35e-05	2	−2.01e-06	.800	−1.76e-05, 1.36e-05
		3	1.23e-05	.158	−4.79e-06, 2.94e-05	3	1.28e-05	.145	−4.40e-06, 2.99e-05	3	1.27e-05	.146	−4.44e-06, 2.99e-05
		4	1.06e-06	.908	−1.69e-05, 1.88e-05	4	1.70e-06	.852	−1.62e-05, 1.96e-05	4	1.66e-06	.856	−1.63e-05, 1.96e-05

*Notes*: Significant associations are in bold. Model 1: raw model. Model 2: adjusted by age, sex, BMI, MAPT randomization group, and educational level. Model 3: Model 2 + plasma IL-6 levels. CI = confidence interval; CCS = composite cognitive score; MMSE = Mini-Mental State Examination, FCSRT = Free and Cued Selective Reminding Test; BMI = body mass index; MAPT = Multidomain Alzheimer Preventive Trial.

### Associations Between Periostin and Capacities (Periostin as Categorical)

In the categorical approach, the POSTN+ group, compared to the POSTN− group, showed a greater decline in GS and the SPPB score at every 1-year-apart visit (β = −0.057, 95% CI = 0.101, −0.013 and β = −0.736, 95% CI = −1.091, −0.381 at the 4-year follow-up visit, respectively) in full-adjusted models. In addition, POSTN+ displayed a significantly poorer performance in the 5-STS only in the last time-point (β = 1.681, 95% CI = 0.801, 2.561). No differences in evolution were found in the HS (Table 4). As displayed in Table 5, among the cognitive capacities, we found significant differences in the CCS score along the 4 years (worse evolution in the periostin+ group, β = −0.215, 95% CI = −0.335, −0,094). Models without IL-6 as a covariate (Model 2) yielded virtually the same results (Supplementary Tables S2–S4).

**Table 4.** Full-Adjusted Associations Between Periostin and Longitudinal Evolution in Physical Capacities (Periostin as Categorical)

	Low Plasma Periostin		High Plasma Periostin*		Between-Group Difference		
	Within-Group Evolution Estimated Mean (95% CI)	*p*-Value	Within-Group Evolution Estimated Mean (95% CI)	*p*-Value	Estimated Difference (95% CI)	*p*-Value	*p* for Trend
	Gait speed (m/s),* n* = 1075						
1	−0.023 (−0.039, −0.006)	.008	−0.091 (−0.125, −0.057)	<.001	**−0.068 (−0.103, −0.034)**	**<.001**	**.004**
2	−0.075 (−0.093, −0.058)	<.001	−0.127 (−0.163, −0.092)	<.001	**−0.052 (−0.089, −0.016)**	**.005**	
3	−0.075 (−0.944, −0.055)	<.001	−0.129 (−0.168, −0.089)	<.001	**−0.054 (−0.095, −0.012)**	**.011**	
4	−0.076 (−0.097, −0.056)	<.001	−0.134 (−0.175, −0.092)	<.001	**−0.057 (−0.101, −0.013)**	**.011**	
	SSPB score,* n* = 1071						
1	−0.126 (−0.241, −0.012)	.031	−0.438 (−0.685, −0.192)	<.001	**−0.312 (−0.563, −0.613)**	**.015**	**<.001**
2	−0.275 (−0.395, −0.154)	<.001	−0.555 (−0.821, −0.289)	<.001	**−0.280 (−0.555, −0.005)**	**.045**	
3	−0.282 (−0.424, −0.139)	<.001	−0.838 (−1.141, −0.536)	<.001	**−0.557 (−0.878, −0.235)**	**<.001**	
4	−0.476 (−0.631, −0.0321)	<.001	−1.212 (−1.543, −0.881)	<.001	**−0.736 (**−**1.091, −0.381)**	**<.001**	
	STS test (s),* n* = 1062						
1	0.38 (0.08, 0.68)	.012	0.22 (−0.36, 0.81)	.454	−0.155 (−0.755, 0.444)	.611	**<.001**
2	0.28 (−0.03, 0.59)	.077	0.56 (−0.08, 1.20)	.085	0.277 (−0.384, 0.939)	.411	
3	0.44 (0.07, 0.80)	.020	0.45 (−0.28, 1.19)	.229	0.016 (−0.768, 0.801)	.967	
4	0.59 (0.19, 0.99)	.004	2.27 (1.45, 3.08)	<.001	**1.681 (0.801, 2.561)**	**<.001**	
	Handgrip strength,* n* = 1069						
1	−1.28 (−1.70, −0.85)	<.001	−2.04 (−3.00, −1.07)	<.001	−0.76 (−1.74, 0.22)	.127	.563
2	−2.52 (−2.95, −2.09)	<.001	−2.99 (−3.99, −2.01)	<.001	−0.48 (−1.48, 0.52)	.350	
3	−4.14 (−4.61, −3.66)	<.001	−5.19 (−6.25, −4.14)	<.001	−1.06 (−2.14, 0.03)	.057	
4	−5.38 (−5.88, −4.89)	<.001	−5.77 (−6.87, −4.67)	<.001	−0.38 (−1.52, 0.76)	.510	

*Notes*: Significant associations are in bold. Models were adjusted by age, sex, BMI, MAPT randomization group, educational level, and plasma IL-6 levels. CI = confidence interval; m/s = meters per second; SPPB = short physical performance battery; STS = sit-to-stand test; kg = kilograms; BMI = body mass index; MAPT = Multidomain Alzheimer Preventive Trial.

*High plasma periostin defined as values in the fourth quartile (>28 169 pg/mL).

**Table 5.** Full-Adjusted Associations Between Periostin and Longitudinal Evolution in Mental Capacities (Periostin as Categorical)

	Low Plasma Periostin *N*=822		High Plasma Periostin* N=274		Between-Group Difference		
	Within-Group Evolution Estimated Mean (95% CI)	*p*-Value	Within-Group Evolution Estimated Mean (95% CI)	*p*-Value	Estimated Difference (95% CI)	*p*-Value	*p* for Trend
	Cognitive composite score,^†^* n* = 1068						
1	−0.093 (−0.122, −0.064)	<.001	−0.269 (−0.366, −0.172)	<.001	**−0.176 (−0.274, −0.078)**	**<.001**	**.029**
2	−0.093 (−0.125, −0.062)	<.001	−0.246 (−0.348, −0.145)	<.001	**−0.153 (−0.256, −0.050)**	**<.001**	
3	−0.187 (−0.223, −0.149)	<.001	−0.351 (−0.459, −0.243)	<.001	**−0.164 (−0.277, −0.052)**	**.004**	
4	−0.222 (−0.263, −0.181)	<.001	−0.437 (−0.551, −0.322)	<.001	**−0.215 (−0.335, −0.094)**	**<.001**	
	Mini-Mental State Examination Score,* n* = 1070						
1	−0.132 (−0.254, −0.009)	.035	−0.197 (−0.464, 0.068)	.146	−0.066 (−0.335, 0.204)	.633	.938
2	−0.156 (−0.282, −0.030)	.015	−0.239 (0.517, −0.039)	.091	−0.083 (−0.366, 0.199)	.565	
3	−0.254 (−0.396, −0.113)	<.001	−0.378 (−0.678, −0.078)	.013	−0.124 (−0.436, 0.188)	.436	
4	−0.231 (−0.379, −0.083)	<.001	−0.413 (−0.727, −0.099)	.010	−0.182 (−0.511, 0.147)	.278	

*Notes*: Significant associations are in bold. Models were adjusted by age, sex, BMI, MAPT randomization group, educational level, and plasma IL-6 levels. CI = confidence interval; CCS = composite cognitive score; MMSE = Mini-Mental State Examination; BMI = body mass index; MAPT = Multidomain Alzheimer Preventive Trial.

*High plasma periostin defined as values in the fourth quartile (>28 169 pg/mL).

^†^Based on the mean *Z*-score of 4 cognitive tests (free and total recall of the Free and Cued Selective Reminding test; 10 MMSE orientation items; Digit Symbol Substitution Test; and Category Naming Test).

## Discussion

The present study shows significant associations between plasma POSTN and the evolution in physical and cognitive functions among community-dwelling older (70 years old and older) adults. Our results show worse performance in the CCS and GS with increasing levels of plasma POSTN at the cross-sectional level, as well as steeper declines in both physical (SPPB score and 5-STS) and cognitive (CCS) capacities in our population. Consistently, when participants are categorized into high- and low-POSTN groups, those in the upper quartile show a worse evolution at both the physical (GS, SPPB score, 5-STS tests) and cognitive (CCS) domain levels.

Nevertheless, due to the lack of previous studies exploring associations between POSTN and the evolution of aging-related functional decline, a causative role remains putative. The biological basis supporting POSTN as a marker of aging arise from its involvement in ECM overproduction and fibrosis (40), the alterations in POSTN paracrine activity (22) as well as its association with inflammation (41).

Healthy functions are achieved via the maintenance of adequate and optimized relationships between the tissue architecture and function. This interplay is the result of bidirectional and permanent interactions between two cell compartments, parenchyma, and stroma (7). In the physiological state, mesenchymal stromal cells (mainly resident fibroblasts) produce, remodel and give support to the ECM to meet tissue functional needs in response to signals that promote ECM structural proteins and MPs production (42). Nevertheless, aging influences the composition, topography, and biomechanics of the ECM, contributing to abnormal cell communication and dysregulated behavior, ultimately leading to organ (parenchymal) dysfunction (43). Additionally, tissue aging is characterized by the excessive accumulation of senescent cells at multiple-system levels, showing the arrest of cell cycle and resistance to apoptosis (44). In this state, both supportive and parenchymal cells acquire a proinflammatory state, known as senescence-associated secretory phenotype (SASP) (45), and their secretome (composed of cytokines, chemokines, mitogenic factors, and proteases) can lead to deleterious effects on surrounding and distant tissues, including inflammation, further senescence, regenerative processes arrest and cell misfunctioning and death (40,46), ultimately affecting whole-body organ failure, that contributes to functional decline and the so-called age-related diseases (47,48).

Although exact mechanisms that result in excessive ECM deposition and accumulation remain scarcely understood, there is increasing evidence linking cellular senescence and fibrosis (26,29). POSTN might play a fundamental role in this process, because it has been shown to up-regulate tumor growth factor-β (TGF-β), a SASP factor that promotes cellular senescence (49,50), which in turn stimulates POSTN production in a positive feedback (41), contributing to further fibrosis and cellular senescence (51), as observed in cardiac, kidney, muscle, and lung tissues from mice (14,26,52).

Therefore, POSTN might constitute a marker of a transversal element of aging, the senescence-related ECM dysfunction, which putatively affects both multiorgan function and communication (7,40), that might manifest itself as physical and cognitive functional declines, previously linked to cell senescence. Our study supports this role by showing an association between blood POSTN levels and physical and cognitive functions, as expressions of multisystemic physiological declines. Nevertheless, given the novelty of the topic, future mechanistic research should describe how POSTN might contribute to tissue aging at the individual system levels and how predictive ability could be improved by its combination with other biomarkers. In addition, population-based studies are needed to shed further light on the potential role of plasma POSTN and other MPs (such as osteopontin (53) and thrombospondin (54), tightly related to age-related fibrosis process) as transversal markers of healthy/accelerated aging.

### Strengths and Limitations

Our study presents strengths such as the relatively large sample size, its novelty, given that it is the first study exploring plasma POSTN associations with functional markers of aging. Furthermore, its longitudinal nature with a relatively long follow-up and several time-points of data collection allows us to know the trajectories of the different physical and cognitive function outcome measures and clearly identify POSTN as an innovative and predictive biomarker of physical and cognitive domain decline.

Nevertheless, our findings should be interpreted considering several limitations. We included a group of relatively healthy and highly educated older adults who participated in a randomized clinical trial; therefore, results may differ in observational studies with demographically diverse populations, and generalization of results should be cautious. However, the interventions of the MAPT study did not have significant effects on cognitive function (33); moreover, all our analyses were adjusted to MAPT group allocation, minimizing potential bias. In fact, the use of MMSE as an endpoint in our study could explain the lack of association between periostin and cognitive evolution (as measured by this instrument) in our study. The inability of MMSE to capture subtle changes in the upper scoring limits might have hampered the possibility of capturing actual associations found when CCS was used as the cognitive outcome, due to greater levels of cognitive reserve in our highly educated population. Attrition (*n* = 101, 9.2% of included participants) might be an issue in our study, given that dropping-out participants might have experienced sharper declines in function that might have gone unnoticed and led to attenuation of effects. In addition, we lacked longitudinal measurements of POSTN, which impeded us to explore concurrent changes in plasma POSTN and functions and from better characterizing the use of POSTN to monitor healthy aging. Moreover, to what extent peripheral POSTN reflects expression in different tissues is not established. Finally, our study should be deemed exploratory, given that potentially, the incorporation of other markers would allow us to better interpret the predictive role of POST as a marker of aging

## Conclusion

The current study showed for the first time that higher plasma POSTN levels were associated with declines in both physical and cognitive function performance in older adults over a 4-year follow-up. Given the novelty of the topic, further mechanistic and population-based research is guaranteed to explore mechanisms underlying the associations between POSTN and functional trajectories of aging, as well as the validity of this MP as a marker of functional decline at an older age.

## Supplementary Material

glac226_suppl_Supplementary_Tables

## Data Availability

The data that support the findings of this study are available from MAPT Study Group, but restrictions apply to the availability of these data, which were used under license for the current study, and so are not publicly available. Data are however available from the authors upon reasonable request and with permission of Nicola Coley (nicola.coley@inserm.fr).
