# Supplementary material for: Periostin Plasma Levels and Changes on Physical and Cognitive Capacities in Community-Dwelling Older Adults
Source: J Gerontol A Biol Sci Med Sci. 2022 Nov 14;78(3):424–32. doi: 10.1093/gerona/glac226 (PMC9977245; doi:10.1093/gerona/glac226)
Supplement: glac226_suppl_Supplementary_Tables [file glac226_suppl_supplementary_tables.docx]

**Supplementary Table S1.Baseline characteristics of included vs. non-included subjects in the present analyses**

| **Characterstics** | Whole Sample  Total (n=1679) | Non-included  (n=583) | High Included  (n=1096)^¥^ |
| --- | --- | --- | --- |
| **Women, No. (%)** | 700 (63.87%) | 554 (67.40) | 146 (53.28) |
| **Age, y** | 75.34 (4.42) | 75.39 (4.53) | 75.31 (4.37) |
| **MAPT group allocation, No. (%)** |  |  |  |
| Omega 3 + MDI group | 417 (24.84%) | 143 (24.3%) | 274 (25%) |
| Omega 3 group | 422 (25.13%) | 156( 26.76%) | 266 (24.27%) |
| MDI group | 420 (25.01%) | 143 (24.53%) | 277 (25.27%) |
| Control group | 420 (25.01%) | 141 (24.19%) | 279 (25.46%) |
| **Education, No. (%)** |  |  |  |
| No diploma | 85 (5.17%) | 36 (6.38%) | 49 (4.54%) |
| Primary school certificate | 286 (17.41%) | 108 (19.15%) | 178 (16.50%) |
| Secondary education | 553 (33.66%) | 199 (35.28%) | 354 (32.81%) |
| High school diploma | 242 (14.73%) | 74 (13.12%) | 168 (15.57%) |
| University level | 477 (29.03%) | 147 (26.06%) | 330 (30.58%) |
| **Body Mass Index** ^ⴕ^ | 26.27 (4.04) | 26.46 (3.99) | 26.22 (4.05) |
| **Gait speed (m/s)** | 1.09 (0.26) | 1.07 (0.24) | 1.10 (0.27) |
| **SPPB score** | 10.62 (1.77) | 10.41 (1.83) | 10.68 (1.75) |
| **SPPB- STS test (s)** | 11.64 (3.85) | 12.08 (4.03) | 11.51 (3.79)* |
| **Handgrip strength (kg)** | 26.67 (9.79) | 26.46 (9.98) | 26.73 (9.74) |
| **CCS**^‡^ | 0.01 (0.69) | -0.05 (0.72) | 0.03 (0.69)* |
| **MMSE** | 28.03 (1.88) | 27.89 (2.11) | 28.07 (1.81) |
| **FCSRT Delayed Recall** | - 1. (10.57) | 75.07 (10.69) | 76.11 (10.53) |

Abbreviations: MDI=Multi-Domain Intervention; MAPT= Multidomain Alzheimer Preventive Trial; m/s: meters per second; SPPB: Short Physical Performance Battery; STS: Sit-to-stand Test; kg=kilograms; CCS: Composite Cognitive Score; MMSE=Mini-Mental State Examination, FSRT: Free and Cued Selective Reminding Test.

¥. Subjects were included if data for the purpose of this study was present at the 1-year MAPT study visit

ⴕ. Body mass index calculated as weight in kilograms divided by height in meters squared.

‡. Based on the z score of 4 cognitive tests (free and total recall of the Free and Cued Selective Reminding test, 10 MMSE orientation items, Digit Symbol Substitution Test, and Category Naming Test).

*P <0.05 based on Student T-test test or Pearson χ2 test (between periostin groups)

**Supplementary Table 2. Associations between periostin and longitudinal evolution in mental and physical capacities (periostin as continuous)**

|  |  |  | **Baseline periostin levels** | | | **Time** | | | **Baseline periostin x time** | | |
| --- | --- | --- | --- | --- | --- | --- | --- | --- | --- | --- | --- |
|  |  | Sample size | Coefficient | p | 95%CI | Coefficient | p | 95%CI | Coefficient | p | 95%CI |
| **Gait speed (m/s)** | Model 1 | 1096 | **-2.75e-06** | **0.017** | **-5.01e-06,-4.89e-07** | **-0.01** | **0.001** | **-0.02, -0.004** | 1.95e-08 | 0.894 | -2.67e-07, 3.06e-07 |
|  | Model 2 | 1075 | **-3.16e-06** | **0.007** | **-5.43e-06, -8.77e-07** | **-0.01** | **0.001** | **-0.02, -0.005** | 4.48e-08 | 0.758 | -2.40e-07, 3.30e-07 |
| **SPPB score** | Model 1 | 1092 | -7.20e-06 | 0.366 | -0.23e-05, 8,40e-06 | -0.003 | 0.911 | -0.06, 0.05 | **-2.57e-06** | **0.022** | **-4.77e-06, -3.73e-07** |
|  | Model 2 | 1071 | -9.07e-06 | 0.254 | -0.24e-05, 6.52e-06 | -0.003 | 0.895 | -0.059, 0.051 | **-2.56e-06** | **0.023** | **-4.77e-06, -3.57e-07** |
| **SPPB- STS test (s)** | Model 1 | 1083 | **-3.8e-05** | **0.046** | **-0.76e-05, -6.98e-07** | -0.091 | 0.210 | -0.234, 0.051 | **8.03e-06** | **0.006** | **2.29e-06, 1.38e-05** |
|  | Model 2 | 1062 | -3-14e-05 | 0.106 | -6.95e-05, 6.66e-06 | -0.085 | 0.248 | -0.228, 0.059 | **7.68e-06** | **0.009** | **1.93e-06, 1.34e-05** |
| **Handgrip strength (kg)** | Model 1 | 1090 | -4.91e-05 | 0.122 | -1.11e-04, 1.31e-05 | **-0.724** | **<0.001** | **-0.897, -0.551** | 2.04e-04 | 0.565 | -4.91e-06, 8.99e-06 |
|  | Model 2 | 1069 | -4.26e-05 | 0.182 | 1.05e-04, 0.2e-05 | **-0.716** | **<0.001** | **-0.892, -0.541** | 1.73 e-06 | 0.628 | -5.28e-06, -8.74e-06 |
| **CCS** | Model 1 | 1088 | **-1.02e-05** | **0.001** | **-1.61e-05, -4.31e-06** | -0.013 | 0.080 | -0.028, 0.002 | **-6.41e-07** | **0.033** | **-1.23e-06, -5.25e-08** |
|  | Model 2 | 1068 | **-1.05e-05** | **<0.001** | **-1.62e-05, -4.81e-06** | -0.014 | 0.073 | -0.028, 0.001 | **-6.35e-07** | **0.039** | **-1.24e-06, -3.36e-08** |
| **MMSE** | Model 1 | 1090 | -1.4e-05 | 0.111 | -3.13e-06, 3.23 e-06 | -0.033 | 0.196 | -0.085, 0.017 | -1.19e-07 | 0.910 | -1.93e-06, 2.17e-06 |
|  | Model 2 | 1070 | -1.5e-05 | 0.084 | -3.21e-05, 2.01e-06 | -0.035 | 0.180 | -0.087, 0.016 | 2.36e-07 | 0.822 | -1.82e-06, 2.29e-06 |
| **FCSRT** | Model 1 | 1089 | -6.32e-05 | 0.187 | -1.56e-04, 3.06e-05 | -0.041 | 0.748 | -0.289, 0.208 | **-1.34e-05** | **0.008** | **-2.33e-05, -3.42e-06** |
|  | Model 2 | 1069 | -6.68e-05 | 0.162 | -1.6e-04, 2.69e-05 | -0.051 | 0.691 | -0.304, 0.201 | **-1.33e-05** | **0.010** | **-2.34e-05, -3.19e-06** |

Abbreviations: CI=Confindence Interval; m/s: meters per second; SPPB: Short Physical Performance Battery; STS: Sit-to-stand Test; kg=kilograms; CCS: Composite Cognitive Score; MMSE=Mini-Mental State Examination, FSRT: Free and Cued Selective Reminding Test.

**Supplementary Table 3. Associations between periostin and longitudinal evolution in physical capacities (periostin as categorical)-Model 2**

|  | **Low plasma periostin** | | | **High plasma periostin** | | **Between-group Difference** | |  |
| --- | --- | --- | --- | --- | --- | --- | --- | --- |
|  | **Within-group evolution**  **Estimated mean**  **(95% CI)** | | **p-value** | **Within-group evolution**  **Estimated mean**  **(95% CI)** | **p-value** | **Estimated difference**  **(95%CI)** | **p-value** | **p for trend** |
|  | | **Gait Speed (m/s), n=1075** | | | | | |  |
| 12 months | -0.023 (-0.039, -0.006) | | 0.008 | -0.091 (-0.125, -0.057) | <0.001 | -0.068 (-0.103, -0.034) | **<0.001** | **0.003** |
| 24 months | -0.075 (-0.093, -0.058) | | <0.001 | -0.127 (-0.163, -0.092) | <0.001 | -0.052 (-0.089, -0.016) | **0.005** |  |
| 36 months | -0.075 (-0.944, -0.055) | | <0.001 | -0.129 (-0.168, -0.089) | <0.001 | -0.054 (-0.095, -0.012) | **0.011** |  |
| 48 months | -0.076 (-0.097, -0.056) | | <0.001 | -0.134 (-0.175, -0.092) | <0.001 | -0.057 (0.101, -0.013) | **0.011** |  |
|  | | **SSPB score, n=1071** | | | | | |  |
| 12 months | -0.126 (-0.241, -0.012) | | 0.031 | -0.438 (-0.685, -0.192) | <0.001 | -0.312 (-0.563, -0.613) | **0.015** | **<0.001** |
| 24 months | -0.275 (-0.395, -0.154) | | <0.001 | -0.555 (-0.821, -0.289) | <0.001 | -0.280 (-0.555, -0.005) | **0.045** |  |
| 36 months | -0.282 (-0.424,-0.139) | | <0.001 | -0.838 (-1.141, -0.536) | <0.001 | -0.557 (-0.878, -0.235) | **<0.001** |  |
| 48 months | -0.476 (-0.631, -0.0321) | | <0.001 | -1.212 (-1.543, -0.881) | <0.001 | -0.736 (-1.091, -0.381) | **<0.001** |  |
|  | | **SPPB-STS test (s), n= 1062** | | | | | |  |
| 12 months | 0.38 (0.08, 0.68) | | 0.012 | 0.22 (-0.36, 0.81) | 0.454 | -0.155 (-0.755, 0.444) | 0.611 | **<0.001** |
| 24 months | 0.28 (-0.03, 0.59) | | 0.077 | 0.56 (-0.08, 1.20) | 0.085 | 0.277 (-0.384, 0.939) | 0.411 |  |
| 36 months | 0.44 (0.07, 0.80) | | 0.020 | 0.45 (-0.28, 1.19) | 0.229 | 0.016 (-0.768, 0.801) | 0.967 |  |
| 48 months | 0.59 (0.19, 0.99) | | 0.004 | 2.27 (1.45, 3.08) | <0.001 | 1.681 (0.801, 2.561) | **<0.001** |  |
|  | | **Handgrip Strength (kg), n=1069** | | | | | |  |
| 12 months | -1.28 (-1.70, -0.85) | | <0.001 | -2.04 (-3.00, -1.07) | <0.001 | -0.76 (-1.74, 0.22) | 0.127 | 0.514 |
| 24 months | -2.52 (-2.95, -2.09) | | <0.001 | -2.99 (-3.99, -2.01) | <0.001 | -0.48 (-1.48, 0.52) | 0.350 |  |
| 36 months | -4.14 (-4.61, -3.66) | | <0.001 | -5.19 (-6.25, -4.14) | <0.001 | -1.06 (-2.14, 0.03) | 0.057 |  |
| 48 months | -5.38 (-5.88, -4.89) | | <0.001 | -5.77 (-6.87, -4.67) | <0.001 | -0.38 (-1.52, 0.76) | 0.510 |  |

Abbreviations: CI=Confindence Interval; m/s: meters per second; SPPB: Short Physical Performance Battery; STS: Sit-to-stand Test; kg=kilograms;

**Supplementary Table 4. Associations between periostin and longitudinal evolution in mental capacities (periostin as categorical)-Model 2**

|  | **Low plasma periostin** | | | **High plasma periostin** | | **Between-group Difference** | | |
| --- | --- | --- | --- | --- | --- | --- | --- | --- |
|  | **Within-group evolution**  **Estimated mean**  **(95% CI)^c^** | | **p-value** | **Within-group evolution**  **Estimated mean**  **(95% CI)** | **p-value** | **Estimated difference**  **(95%CI)** | **p-value** | **P for trend** |
|  | | **Cognitive Composite Score , n=1068** | | | | | |  |
| 12 months | -0.095 (-0.124, -0.065) | | <0.001 | -0.248 (-0.342, -0.154) | <0.001 | -0.153 (-0.249, -0.058) | **0.001** | **0.027** |
| 24 months | -0.097 (-0.128, -0.065) | | <0.001 | -0.223 (-0.323, -0.125) | <0.001 | -0.127 (-0.228, -0.026) | **0.013** |  |
| 36 months | -0.189 (-0.226, -0.152) | | <0.001 | -0.332 (-0.438, -0.226) | <0.001 | -0.142 (-0.253, -0.032) | **0.011** |  |
| 48 months | -0.226 (-0.267, -0.184) | | <0.001 | -0.422 (-0.534, -0.308) | <0.001 | -0.196 (-0.315, -0.076) | **0.001** |  |
|  | | **Mini-Mental State Examination Score, n=1070** | | | | | |  |
| 12 months | -0.133 (-0.258, -0.009) | | 0.036 | -0.154 (-0.416, 0.107) | 0.146 | -0.066 (-0.335, 0.204) | 0.875 | 0.926 |
| 24 months | -0.148 (-0.276, -0.020) | | 0.023 | -0.176 (-0.448, 0.097) | 0.091 | -0.083 (-0.366, 0.199) | 0.845 |  |
| 36 months | -0.248 (-0.391, -0.105) | | 0.001 | -0.378 (-0.616, -0.025) | 0.013 | -0.124 (-0.436, 0.188) | 0.645 |  |
| 48 months | -0.223 (-0.373, -0.074) | | 0.003 | -0.413 (-0.658, -0.040) | 0.010 | -0.182 (-0.511, 0.147) | 0.448 |  |
|  | | **Free and Cued Selective Reminding Test, n= 1069** | | | | | |  |
| 12 months | -2.12 (-2.61, -1.65) | | <0.001 | -3.19 (-4.74, -1.64) | <0.001 | -1.07 (-2.63, 0.50) | 0.182 | 0.108 |
| 24 months | -1.44 (-1.96, -0.92) | | <0.001 | -1.99 (-3.62, -0.36) | 0.009 | -0.55 (-2.22, 1.12) | 0.519 |  |
| 36 months | -2.86 (-3.48, -2.23) | | <0.001 | -4.47 (-6.24, -2.71) | <0.001 | -1.62 (-3.46, 0.22) | 0.085 |  |
| 48 months | -2.91 (-3.61, -2.21) | | <0.001 | -5.18 (-7.07, -3.29) | <0.001 | -2.27 (-4.27, -0.28) | **0.026** |  |

Abbreviations: CI=Confindence Interval
